# Impaired serum neutralization and death in Omicron-infected critically ill patients: insights from the French SEVARVIR prospective, multicenter cohort study

**DOI:** 10.1186/s40635-025-00831-y

**Published:** 2025-11-26

**Authors:** Timothée Bruel, Isabelle Staropoli, Pierre Bay, Paul Bastard, Sébastien Préau, Aurélie Guigon, Antoine Guillon, Karl Stefic, Fabrice Uhel, Stéphane Pelleau, Laura Garcia, Anne Puel, Aurélie Cobat, Jean-Laurent Casanova, Jean-Michel Pawlotsky, Michael White, Olivier Schwartz, Slim Fourati, Nicolas de Prost

**Affiliations:** 1 Antiviral Activities of Antibodies Group, Institut Pasteur, Université Paris Cité, CNRS UMR3569, Paris, France; 2 Virus and Immunity Unit, Institut Pasteur, Université Paris Cité, CNRS UMR3569, 25–28 Rue du Docteur Roux, 75015 Paris, France; 3 Vaccine Research Institute, Créteil, France; 4 Service de Médecine Intensive Réanimation, DMU Médecine, Hôpitaux Universitaires Henri Mondor, AP-HP (Assistance Publique-Hôpitaux de Paris), 1, Rue Gustave Eiffel, 94010 Créteil, France; 5 GRC CARMAS, Faculté de Santé de Créteil, UPEC (Université Paris-Est-Créteil), 94010 Créteil, France; 6 Université Paris-Est-Créteil (UPEC), Créteil, France; 7 IMRB INSERM U955, Team “Viruses, Hepatology, Cancer”, Créteil, France; 8 St. Giles Laboratory of Human Genetics of Infectious Diseases, Rockefeller Branch, The Rockefeller University, New York, NY USA; 9 Laboratory of Human Genetics of Infectious Diseases, Necker Branch, INSERM U1163, Necker Hospital for Sick Children, Paris, France; 10 University of Paris, Imagine Institute, Paris, France; 11 Pediatric Hematology-Immunology and Rheumatology Unit, Necker Hospital for Sick Children, Assistante Publique-Hôpitaux de Paris (AP-HP), Paris, France; 12 U1167-RID-AGE Facteurs de Risque et Déterminants Moléculaires des Maladies Liées au Vieillissement, University Lille, Inserm, CHU Lille, Institut Pasteur de Lille, 59000 Lille, France; 13 Service de Virologie, CHU Lille, Lille, France; 14 Service de Médecine Intensive Réanimation, CHU Tours, Université de Tours, Tours, France; 15 Inserm U1100, Centre d’Etudes Des Pathologies Respiratoires (CEPR), Tours, France; 16 Service de Virologie, CHU Tours, Université de Tours, Tours, France; 17 Université Paris Cité, INSERM UMR-S1151, CNRS UMR-S8253, Institut Necker-Enfants Malades, 75015 Paris, France; 18 Médecine Intensive Réanimation, AP-HP, Hôpital Louis Mourier, DMU ESPRIT, 92700 Colombes, France; 19 Infectious Disease Epidemiology and Analytics G5 Unit, Institut Pasteur, Université Paris Cité, Paris, France; 20 Howard Hughes Medical Institute, New York, NY USA; 21 Department of Virology, Hôpitaux Universitaires Henri Mondor, Assistance Publique–Hôpitaux de Paris, Créteil, France

**Keywords:** COVID-19, ICU, Neutralization, Variants

## Abstract

**Background:**

Despite advances in treatment, critically ill COVID-19 patients requiring intensive care unit (ICU) admission continue to comprise a substantial proportion of cases. However, the factors influencing poor prognosis in this population remain poorly understood. To address this knowledge gap, we conducted a prospective analysis of serum neutralizing activity against SARS-CoV-2 in 49 non-selected, critically ill COVID-19 patients enrolled in the multicenter SEVARVIR cohort between October 2022 and May 2024.

**Methods:**

This a substudy of the SEVARVIR prospective multicenter observational cohort study (NCT05162508). We included 49 critically ill COVID-19 patients hospitalized in four French intensive care units between October 2022 and May 2024 from the 827 patients enrolled in the multicenter, prospective SEVARVIR study. Serum neutralizing titers of authentic SARS-CoV-2 isolates were measured using the S-Fuse assay and patients categorized as neutralizers if they had an anti-spike serum neutralization titer against the infecting variant > 15 and non-neutralizers if ≤ 15. Full-length SARS-CoV-2 genomes from all included patients were sequenced by means of next-generation sequencing.

**Results:**

Median age was 73 years (59–75) and 34.7% of patients (*n* = 17/49) were female. Half of the patients (53.1%, *n* = 26/49) had immunosuppressive comorbidities. A large proportion of individuals lacked the capacity to neutralize their infecting variant (57.1%, *n* = 28/49). Neutralizing titers were significantly higher in 28-day survivors than in deceased patients (p = 0.0212) and neutralizers had a significantly lower 28-day mortality than non-neutralizers (5.0%, *n* = 1/21 vs. 32.1%, *n* = 9/28; p = 0.0312). Nine out of the ten patients who succumbed to the disease within 28 days of admission had undetectable serum neutralizing capacity, which was significantly more prevalent than in survivors (*p* = 0.03), irrespective of immunosuppression status. The sole patient who died despite having detectable neutralizing antibodies against SARS-CoV-2, was found to have anti-interferon auto-antibodies.

**Conclusion:**

These findings underscore the potential benefits of early therapeutic interventions aimed at enhancing neutralization, which may improve survival outcomes in both immunocompetent and immunocompromised critically ill COVID-19 patients.

**Supplementary Information:**

The online version contains supplementary material available at 10.1186/s40635-025-00831-y.

## Background

Protection against SARS-CoV-2 infection and severe COVID-19 has been linked to neutralizing antibodies [1]. Measuring serum neutralization is used as a correlate of protection for COVID-19 and enables immunobridging studies [2]. This is mechanistically explained by the capacity of neutralizing antibodies to prevent infection and provide sterilizing immunity. It is believed that direct antiviral inhibition becomes increasingly unnecessary as the disease progresses due to the growing contribution of immunological mechanisms. Indeed, the most extreme form of COVID-19, life-threatening COVID-19 pneumonia with the acute respiratory distress syndrome, typically develops several days after infection and is primarily driven by a dysregulated immune response, as evidenced by the efficacy of anti-inflammatory therapeutics. The presence of auto-antibodies against type I interferons (AAB-I-IFNs) is a major risk factor, again reinforcing the critical role of the immune response in the physiopathology of severe COVID-19 [3]. Accordingly, patients with severe disease usually produce higher levels of antibodies and progression to the severe form has been associated with a delay in induction of neutralization rather than a lack of their production [4, 5]. However, two recent randomized clinical trials demonstrated the benefit of neutralization (provided by either monoclonal antibodies [6] or convalescent plasma [7]) for patients with severe to critical COVID-19. Optimal antibody-mediated therapeutic efficacy relies on early intervention, a high neutralizing capacity, and an inadequate endogenous antibody response at the time treatment is initiated [8]. Therefore, the impact of neutralization against SARS-CoV-2 on the outcome of patients with critical COVID-19 still requires investigation.

The release of COVID-19 vaccines and the global circulation of SARS-CoV-2 dramatically expanded the proportion of SARS-CoV-2 seropositive individuals. Nevertheless, levels of immunity remain heterogeneous. While only subtle differences exist among mRNA vaccines, all vaccines are not equal in the immune response they elicit [9, 10]. A history of natural infection profoundly reshapes immune response, leading to what is now called “hybrid immunity”, which has been clearly demonstrated to confer the highest level of immune protection [11–13]. While it is accepted that most immunocompetent individuals have immune memory against SARS-CoV-2, the increase of immune-evading variants and waning immunity could reduce neutralizing capacity at the individual level. The impact of this on the outcome of a breakthrough infection is still unclear. This is particularly relevant to the JN.1 family of variants, which emerged in 2023 and displays considerable immune evasion properties.

We tested the hypothesis that critically ill COVID-19 patients infected with the latest Omicron sublineages who had a worse outcome had lower neutralizing antibody titers against SARS-CoV-2 on admission to the intensive care unit (ICU).

## Methods

### Study design and patients

The current study is a substudy of the SEVARVIR prospective multicenter observational cohort study (NCT05162508). We included 49 critically ill COVID-19 patients hospitalized in four French intensive care units (CHU Henri Mondor, Créteil; CHU Louis Mourier, Colombes; CHU de Lille; CHU de Tours) between October 2022 and May 2024 from the 827 patients enrolled in the multicenter, prospective SEVARVIR study since it started enrolling patients (December 2021). Patients were eligible for inclusion in the current analysis if they had had a blood sample drawn within 7 days of ICU admission and if they presented with the following inclusion criteria: age ≥ 18 years, SARS-CoV-2 infection confirmed by a positive reverse transcriptase-polymerase chain reaction in nasopharyngeal swab samples, admission to the ICU for acute respiratory failure (i.e., peripheral oxygen saturation (SpO2) ≤ 90% and need for supplemental oxygen or any kind of ventilator support), patient or next of kin informed of study inclusion. Patients with SARS-CoV-2 infection but no acute respiratory failure or with an RT-PCR cycle threshold (Ct) value > 32 in nasopharyngeal swabs were not included. The study was approved by the Comité de Protection des Personnes Sud-Méditerranée I (N° EudraCT/ID-RCB: 2021-A02914-37). Informed consent was obtained from all patients or their relatives.

Demographics, clinical and laboratory variables were recorded upon ICU admission and during the ICU stay. The severity of the disease upon ICU admission was assessed using the World Health Organization (WHO) 10-point ordinal scale [14] and the Sequential Organ Failure Assessment (SOFA [15]) score. The primary clinical endpoint of the study was day 28 mortality.

### SARS-CoV-2 variant determination

Full-length SARS-CoV-2 genomes from all included patients were sequenced by means of next-generation sequencing as previously described [16]. Full-length viral genome sequence analysis yielding high coverage has been deposited in Genbank (accession numbers: OQ423348, OQ423375, OQ423376, OQ423378, OQ423380, PP357648, PP357634–PP357642, PP357644, PP357646, PP357720, PP357724–PP357728, PP357730, PP357732–PP357735, PP357745, PP357747, PP357749, PP357751, PP357753, PP357758, PP357761, PP357764).

### Seroneutralization

Serum neutralizing titers of authentic SARS-CoV-2 isolates were measured using the S-Fuse assay as previously described [17]. All sera/plasma were heat-inactivated for 30 min at 56 °C. Effective dose 50% (referred to as “titers”), in dilution, was calculated with a reconstructed curve using the percentage of the neutralization at the different concentrations. Neutralization was performed using a D614G strain as control and the corresponding infecting variant (Delta, BQ.1.1, XBB.1.5 or JN.1). The neutralization cut-off titer was set to 30, as it corresponds to the first dilution of serum tested.

### Functional evaluation of anti-interferon auto-antibodies by luciferase reporter assays

Auto-Abs neutralizing type I IFNs positivity was assessed on serum samples collected at ICU admission. The blocking activity of anti-IFN-α2 and anti-IFN-ω auto-Abs was determined with a reporter luciferase activity, as previously described [3]. Briefly, HEK293T cells were transfected with a plasmid containing the Firefly luciferase gene under the control of the human ISRE promoter in the pGL4.45 backbone, and a plasmid constitutively expressing Renilla luciferase for normalization (pRL-SV40). Cells were transfected in the presence of the X-tremeGene9 transfection reagent (Sigma-Aldrich, ref. number 6365779001) for 24 h. Cells in Dulbecco’s modified Eagle medium (DMEM, Thermo Fisher Scientific) supplemented with 2% fetal calf serum (FCS) and 10% healthy control or patient serum (after inactivation at 56 °C, for 20 min) were either left unstimulated or were stimulated with IFN-α2 (Miltenyi Biotec, ref. number 130–108–984), IFN-ω (Merck, ref. number SRP3061), at 10 ng/mL or 100 pg/mL, or IFN-β (Miltenyi Biotec, ref. number: 130-107-888) at 10 ng/mL, for 16 h at 37 °C. Each sample was tested once for each cytokine and dose. Finally, cells were lysed for 20 min at room temperature and luciferase levels were measured with the Dual-Luciferase^®^ Reporter 1000 assay system (Promega, ref. number E1980), according to the manufacturer’s protocol. Luminescence intensity was measured with a VICTOR-X Multilabel Plate Reader (PerkinElmer Life Sciences, USA). Firefly luciferase activity values were normalized against Renilla luciferase activity values. These values were then normalized against the median induction level for non-neutralizing samples, and expressed as a percentage. Samples were considered neutralizing if luciferase induction, normalized against Renilla luciferase activity, was below 15% of the median values for controls tested the same day.

### Luminex

The multiplex serological assay was performed using a previously described [18] panel of antigen-coupled beads encompassing vaccine-preventable diseases and common viral infections (see the full list of the 33 antigens from endemic respiratory viruses and mandatory vaccines in France in Table S1, available in the online supplement). To measure the antibody levels against these antigens, we employed the Intelliflex^®^ instrument from Luminex^®^.

### Statistical analysis

Descriptive results are presented as median (1st–3rd quartiles) for continuous variables, and as numbers with percentages for categorical variables. Two-tailed p-values < 0.05 were considered statistically significant. Patients were categorized as neutralizers if they had an anti-spike serum neutralization titer against the infecting variant > 15 and non-neutralizers if ≤ 15. To avoid multiplicity bias, no other comparisons were performed. Unadjusted comparisons between neutralizers and non-neutralizers were performed using Chi-square or Fisher’s exact tests for categorical variables, and Mann–Whitney tests for continuous variables, as appropriate. Analyses were performed using GraphPad Prism statistical software (v10.4.1) and RStudio software (version 4.2.0). Complex heatmap package [19] was used to generate heatmap plots for visualization.

## Results

**Fig. 1:**
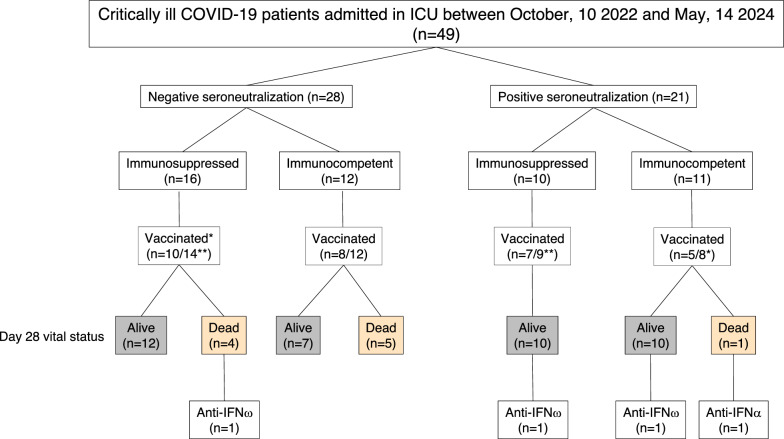
Flowchart of critically ill COVID-19 patients admitted in ICU between October 10 2022 and May, 14 2024 (*n* = 49). *Vaccinated patients had received ≥ 3 vaccine doses; **The vaccination status was unavailable for some patients; anti-IFN: anti-interferon auto-antibodies neutralizing interferon (IFN) *α* or *ω*

Of the 827 patients enrolled in the multicenter, prospective SEVARVIR study (NCT05162508), we included 49 critically ill COVID-19 patients who were hospitalized in four French intensive care units (ICUs) between October 2022 and May 2024. Our primary outcome was mortality on day 28 (Fig. 1). Patients were infected with the following sublineages: XBB (*n* = 21), JN.1 (*n* = 20), BQ.1.1 (*n* = 7), and CH.1.1 (*n* = 1) (Table 1). The median age was 73 years (59–75) and 34.7% of patients (*n *= 17/49) were female. Fifty-three percent of patients (*n* = 26/49) had immunosuppressive comorbidities, including nine who had been exposed to anti-CD20 antibodies (Table S2), while 61.2% (*n* = 30/49) had received at least three vaccine doses. The severity of illness on admission to the ICU was reflected by a median WHO scale score of 6 (IQR 25–75: 6–6), which corresponds to patients requiring high-flow oxygen or non-invasive ventilation support. Two patients received convalescent plasma and two patients received monoclonal antibodies (cilgavimab/tixagevimab; Evusheld), all administered after the blood sample was obtained. Three patients received remdesivir. Four patients (8%) had AAB-I-IFNs. Three neutralized IFN-ω (a member of the Type I interferon family, with ~ 60% sequence similarity to IFN-α) and one neutralized IFN-α2 (one of the most common IFN-α subtypes).

**Table 1:** Characteristics of critically ill COVID-19 patients (*n* = 49)

	All (*n* = 49)
Age, year	73 (59–75)
Gender, female	17 (34.7)
Comorbidities	
Diabetes	12 (24.5)
Chronic heart failure	8 (16.3)
Chronic respiratory failure	11 (22.4)
Chronic renal failure	10 (20.4)
Immunosuppression	26 (53.1)
* Hematological malignancies*	10
* Solid organ transplant*	4
* Solid cancer < 3 years*	5
* Auto-immune disorders*^*1*^	5
* Anti-CD20 monoclonal antibodies*	9
Others^2^	2
Clinical presentation at ICU admission	
First symptoms-ICU admission, days	5 (2–9)
WHO 10-point severity scale	6 (6–6)
SOFA score	4 (2–7)
White blood cell counts, G/L	8.6 (5.1–12.3)
Blood lymphocytes, G/L	0.4 (0.2–0.8)
SARS-CoV-2 vaccination and infection history	
Complete vaccination*	30 (61.2)
Number of doses of vaccine	3 (2–4)
Proven previous SARS-CoV-2 infection	6 (12.2)
Hybrid immunity**	6 (12.2)
SARS-CoV-2 RT-PCR, CT	20 (17–25)
Infecting variant	
CH.1.1	1 (2.0)
BQ.1.1	7 (14.3)
XBB.1.5	21 (42.8)
JN.1	20 (40.8)
Outcomes and management during ICU stay	
Organ failures	22 (44.9)
* Invasive mechanical ventilation*	18 (36.7)
* ECMO support*	0
* Renal replacement therapy*	5 (10.2)
* Vasopressor support*	16 (32.6)
ICU-acquired infection	5 (10.2)
Dexamethasone	29 (59.2)
Tocilizumab	9 (18.4)
Antiviral treatments	
*Convalescent plasma*	2 (4.1)
*Monoclonal antibodies (tixagevimab/cilgavimab)*	2 (4.1)
*Remdesivir*	3 (6.1)
Day 28 mortality	10 (20.4)

Qualitative variables are shown as *n* (%) and continuous variables as median (interquartile range 25–75)

ECMO: extra corporeal membrane oxygenation

^1^Fibrillary glomerulonephritis (*n* = 1), systemic lupus erythematosus (*n* = 1), Anti-glomerular basement membrane antibody disease (*n* = 1), autoimmune hepatitis (*n* = 1), autoinflammatory disease (*n* = 1); ^2^HIV infection (*n* = 1), sickle cell disease (*n* = 1)

^*^3 doses

^**^at least one vaccine dose received and previous SARS-CoV-2 infection

**Fig. 2:**
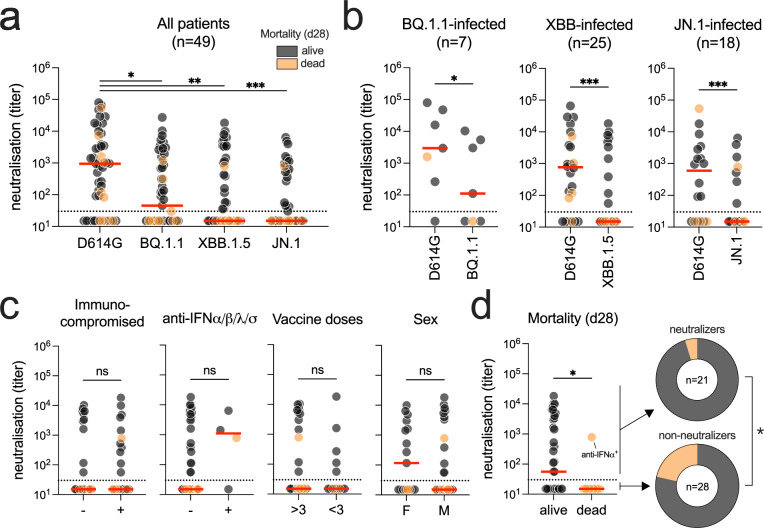
Serum neutralization according to Omicron sublineages and clinical characteristics and outcomes. **a** Neutralizing titers of D614G, BQ.1.1, XBB.1.5 and JN.1 in the sera of critically ill COVID-19 patients (*n* = 49); **b** neutralizing titers according to the variant responsible for the infection in each patient and the D614G strain as control; **c** neutralizing titers against the infected variant across key parameters influencing COVID-19 severity; **d** neutralizing titers against the infecting variant and day 28 mortality. Neutralization was assessed using replicating viral isolates. The dashed line indicates the limit of detection (serum dilution of 30). *P* values come from post hoc comparisons of the Kruskal–Wallis test (**a**), which yielded a global *p* value < 0.001, or Mann–Whitney and Fisher exact tests (**b**, **c**, **d**): ns, not significant, *p < 0.05; **p < 0.01, ***p < 0.001

We measured the capacity of serum samples, collected at ICU admission to neutralize BQ.1.1, XBB.1.5, JN.1, and, as a control, the ancestral strain D614G. Omicron sublineages exhibited lower titers than D614G (Fig. 2a). The median time interval between study inclusion and serum sampling was 0 days (0–3), non-significantly different between non-neutralizers and neutralizers [1 (0–5) vs (0 (0–0); *p* = 0.10)]. When dividing patients according to their specific infecting variant, BQ.1.1-infected individuals displayed slightly higher neutralization than XBB and JN.1-infected patients (111.6 *vs.* 15, for both) (Fig. 2b). A large proportion of individuals (57.1%, *n* = 28/49) lacked the capacity to neutralize their infecting variant. Of note, of the nine patients who had received anti-CD20 antibodies, eight were non-neutralizers (Table S2). None of the other common severity factors were associated with serum neutralization (Fig. 2c and Table 2). Yet, neutralizing titers were significantly higher in 28-day survivors than in deceased patients (*p* = 0.0212 by the Mann–Whitney test), and neutralizers had a significantly lower 28-day mortality than non-neutralizers (5.0%, *n* = 1/21 vs. 32.1%, *n* = 9/28; *p* = 0.0312 by the Fisher exact test) (Fig. 2d and Table 2). Of the ten deceased patients at day 28, nine were unable to neutralize their infecting variant on admission to the ICU. The only one who died despite having detectable neutralizing antibodies against SARS-CoV-2 had AAB-I-IFNs (Figs. 1 and 2d).

**Table 2:** Characteristics of critically ill COVID-19 patients with negative (*n* = 28) or positive (*n* = 21) seroneutralization (infecting variant)

	Negative seroneutralization (*n* = 28)	Positive seroneutralization (*n* = 21)		*P* value
Age, year	73 (63–77)	70 (57–74)		0.23
Gender, female	8 (28.6)	9 (42.8)		0.30
SOFA score	5 (3–8)	3 (2–7)		0.13
Comorbidities				
Diabetes	8 (28.6)	4 (19.0)		0.52
Chronic heart failure	6 (21.4)	2 (9.5)		0.44
Chronic respiratory failure	6 (21.4)	5 (23.8)		> 0.99
Chronic renal failure	7 (25.0)	3 (14.3)		0.48
Immunosuppression*	16 (57.1)	10 (47.6)		0.50
* Hematological malignancies*	8	2		
* Solid organ transplant*	2	2		
* Solid cancer* < *3 years*	3	2		
* Auto-immune disorders*^*1*^	3	2		
* Anti-CD20 in last 12 months*	8 (29)	1 (4.8)		
* Others*^*2*^	0	2		
Vaccination and infection history				
First symptoms-ICU admission, days	4.5 (2.2–8.7)		5 (2.0–9.5)	0.80
Complete vaccination**	18 (69.2)		12 (66.6)	0.85
Number of doses of vaccine	3 (2–4)		3 (2–4)	0.89
Previous SARS-CoV-2 infection	4 (16.6)		2 (11.1)	> 0.99
Hybrid immunity***	4 (16.6)		2 (11.1)	> 0.99
SARS-CoV-2 neutralization titer	< 15		3026 (328–6428)	** < 0.0001**
SARS-CoV-2 RT-PCR, CT	19 (16–22)		21 (18–25)	0.18
Outcomes and management				
Organ failures	14 (50.0)		8 (38.1)	0.41
Invasive mechanical ventilation	11 (39.3)		7 (33.3)	0.67
ICU-acquired infection	4 (14.3)		1 (4.8)	0.37
Dexamethasone	15 (62.5)		14 (77.8)	0.33
Tocilizumab	2 (8.3)		7 (41.2)	**0.021**
Day 28 mortality	9 (32.1)		1 (5.0)	**0.031**

Qualitative variables are shown as n (%) and continuous variables as median (interquartile range 25–75)

^1^Negative seroneutralization: Fibrillary glomerulonephritis (*n* = 1), systemic lupus erythematosus (*n* = 1), Anti–glomerular basement membrane antibody disease (*n* = 1); Positive seroneutralization: autoimmune hepatitis (*n* = 1), autoinflammatory disease (*n* = 1)

^2^HIV infection (*n* = 1), sickle cell disease (*n* = 1)

^*^Patients can have one or more cause of immunosuppression

^**^At least 3 doses received (vaccination status was missing for five patients)

^***^At least one vaccine dose received and previous SARS-CoV-2 infection; *P* values come from Chi-square or Fisher’s exact tests for categorical variables, and Mann–Whitney tests for continuous variables, as appropriate; bolded values are significant at the < 0.05 level. Neutralizing titer is measured against the infecting variant

Among the 28 patients who were non-neutralizers in the serum sample collected upon ICU admission, 13 had a second sample obtained during their ICU stay (drawn after a median time lag of 9 days (IQR 25–75: 4–14) after the first sample). This included eight patients who became neutralizers in the second sample and five who did not. There was a numerical, although not statistically significant, difference in 28-day mortality between patients who became neutralizers and those who did not during their stay in the ICU (12.5% vs 60.0%; *p* = 0.217 by the Fisher’s test).

Among the 28 patients who lacked the capacity to neutralize their infecting variant, 17 had neutralizing antibodies against the ancestral (D614G) variant, while 11 did not. We compared the characteristics and outcomes of patients according to their capacity to neutralize the ancestral variant (Table S3, online supplement). There was no between-group difference regarding clinical characteristics and no association between ancestral variant neutralization and 28-day mortality (day 28 mortality of ancestral variant non-neutralizers vs neutralizers: 30.8%, *n* = 4/13 vs 16.7%, *n* = 6/36; *p* = 0.28).

**Fig. 3:**
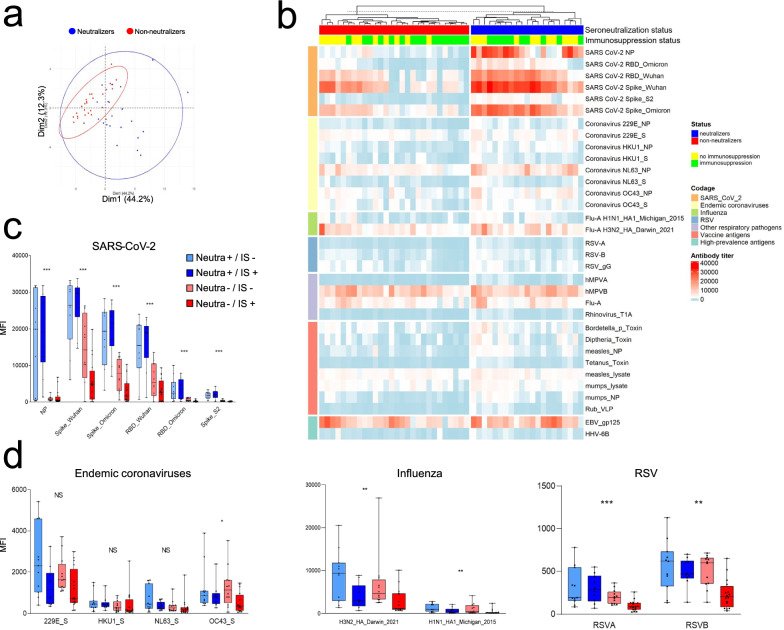
Evaluation of the antibody response against 33 selected antigens. **a** Unsupervised antibody response analysis of the entire cohort depicted by principal component analysis according to the serum neutralizing capacity status; **b** unsupervised heatmap analysis of the antibody titer, illustrated by the serum neutralizing capacity and the immunosuppressive status. The antigens have been sorted according to their nature: SARS-CoV-2 antigens, influenza antigens, respiratory syncytial virus (RSV) antigens, endemic coronaviruses antigens, other respiratory pathogens antigens, vaccine antigens and high-prevalence antigens (light blue indicates low serum antibody titer; dark red indicated high serum antibody titer); **c** antibody titer against selected antigens of SARS-CoV-2, according to the serum neutralizing capacity and the immunosuppressive status; **d** antibody titer against selected antigens of the main other respiratory viruses (influenza, endemic coronaviruses and respiratory syncytial virus (RSV)), according to the serum neutralizing capacity and the immunosuppressive status. The *y*-axis shows antibody titer for each antigen. The data distribution is shown by box plots; each point represents one patient. P-values were calculated using the Kruskal–Wallis test without post hoc test: ns, not significant, *p < 0.05; **p < 0.01, ***p < 0.001

We then sought to determine whether the lack of detectable neutralization against the infecting variant could be explained by a global defect in the humoral response. Using a multiplex Luminex assay, we measured antibody levels against 33 antigens from endemic respiratory viruses and mandatory vaccines in France (Table S1). Principal component analysis showed that the neutralizers and non-neutralizers failed to segregate, indicating that their humoral responses are largely similar (Fig. 3a). A more detailed analysis reveals differences within the group of SARS-CoV-2 antigens, with neutralizers producing the highest levels of antibodies (Fig. 3b and c). Strikingly, antibody levels against SARS-CoV-2 are high within this group of neutralizers, irrespective of immunosuppression status. In contrast, antibody levels against SARS-CoV-2 are strongly influenced by immunosuppression status in the non-neutralizers. The analysis of non-SARS-CoV-2 antigens reveals that antibody levels are better explained by immunosuppression than by neutralizing status (Fig. 3b and d). Overall, these data show that non-neutralizers exhibit reduced antibody responses to SARS-CoV-2 proteins but maintained normal levels (according to their immunological status) for other antigens.

## Discussion

We explored the neutralization capacity of critically ill patients admitted to the ICU for COVID-19-associated acute respiratory failure infected with recent Omicron sublineages. The main findings of our study are as follows: (1) a large proportion of patients lacked the capacity to neutralize their infecting variant; (2) neutralizing titers at baseline were significantly higher in 28-day survivors than in deceased patients; (3) nine out of ten patients who died within 28 days of admission had undetectable serum neutralizing capacity, irrespective of immunosuppression status; and (4) the sole patient who died despite having detectable neutralizing antibodies against SARS-CoV-2, was found to have anti-IFN auto-antibodies.

We observed a strong association between serum neutralization on admission and day 28 mortality, irrespective of immunosuppression status. These findings suggest that rescuing neutralization at ICU admission with monoclonal antibodies or convalescent plasma may benefit both immunocompetent and immunocompromised individuals infected with the latest SARS-CoV-2 variants. In our study, more than half of critically ill COVID-19 patients were unable to neutralize their infecting variant on admission to the ICU. This lack of neutralization capacity was significantly associated with 28-day mortality, but not with clinical variables such as age, gender, vaccination status, and comorbidities, including immunosuppression status. Although the prevalence of immunosuppression did not differ between neutralizers and non-neutralizers, patients who had been exposed to anti-CD20 monoclonal antibodies were more likely to be non-neutralizers, consistent with previous findings [20].

Our findings are consistent with the results of the CONFIDENT trial [7], which demonstrated a significant effect of plasma therapy collected from convalescent donors as compared to standard of care on 28-day mortality in patients with COVID-19 acute respiratory distress syndrome (35.4% vs 45.0%). Only a small proportion of patients in this trial were immunocompromised (hematologic cancer, *n* = 17/475; solid tumor, *n* = 18/475), and our findings suggest that the benefits of convalescent plasma extend beyond patients with known immunosuppressive conditions. A recent multicenter phase three trial conducted in immunocompromised patients with mild COVID-19 randomized patients to receive convalescent plasma plus standard of care vs standard of care alone [21]. The primary outcome was hospitalization for progressive COVID-19 or death at day 28. This study showed a reduction in the primary outcome in patients who received convalescent plasma. Our data, obtained from immunocompromised and immunocompetent patients alike, are therefore consistent with the two aforementioned studies and provide further support for the use of well-characterized, high-titer convalescent plasma in critically ill patients. In January 2025, convalescent plasma was granted a license as a biological product by the United States Food and Drug Administration for use in treating immunocompromised patients with severe SARS-CoV-2 infection. It is worth noting that, while the Infectious Diseases Society of America's guidelines suggest the use of convalescent plasma for ambulatory immunocompromised patients at high risk of progressing to severe disease, they currently recommend against its use for both immunocompetent and immunocompromised hospitalized patients with confirmed COVID-19. Our findings provide evidence to support the idea that maintaining serum neutralization in all critically ill COVID-19 patients (i.e., both immunocompetent and immunocompromised individuals) may be associated with improved survival. Our data also indicate that testing for the presence of anti-IFN auto-antibodies prior to the injection of convalescent plasma may be important.

We found a significant association between the capacity to neutralize the infecting variant and 28-day mortality, but no such association with the ancestral variant. This observation aligns with the significant neutralization escape exhibited by the Omicron variant and its sublineages. Consequently, the presence of neutralizing antibodies against ancestral strains does not guarantee neutralization of the infecting variant. Therefore, adapting viral biomarkers to the specific infecting variant is likely to substantially enhance predictive capacity, as demonstrated here for neutralization.

The Omicron is characterized by the emergence of immune-evading sublineages and waning immunity, in part associated with the accumulation of mutations in the spike protein. This finding highlights the importance of adapting vaccines to circulating variants to ensure effective neutralization of infecting sublineages. It also emphasizes the need to investigate responses to infecting variants rather than prototypical strains (such as the Wuhan of D614G) in physiopathological studies.

Our study has limitations, primarily due to the small number of patients included. This meant that confounding factors, such as exposure to anti-CD20 and tocilizumab, could not be adjusted for in the mortality analysis. As expected, patients with seronegative neutralization were more likely to receive anti-CD20 monoclonal antibodies. More patients with positive seroneutralization were treated with tocilizumab, but this is unlikely to have accounted for the observed difference in mortality between the groups. Indeed, in the Recovery study, the absolute mortality difference between groups was 4% [22]. Similarly, a multivariate analysis could distinguish between neutralization and mortality from severity factors, but this was not feasible here due to the small sample size. Nevertheless, this does not affect our conclusions, since severity factors are balanced across groups. Finally, our study was conducted with a specific set of SARS-CoV-2 variants, which were pooled in the analysis. Therefore, it is possible that their replication potential and immune evasion properties have partly influenced our findings. The strengths of our study include its prospective, multicenter design, which has enabled us to establish a cohort of well-phenotyped patients and explore the most recent Omicron sublineages.

## Conclusions

In this small exploratory study, COVID-19 deaths were associated with a lack of early serum neutralization. Importantly, this lack of neutralization could not be explained by immunosuppression or differences in vaccination. Therefore, differences in vaccine immunogenicity or waning immunity may underlie the lack of neutralization in some immunocompetent individuals. While further work is needed to shed light on the precise mechanism, our data argue in favor of maintaining serum neutralization through repeated vaccination with a vaccine adapted to contemporary sublineages. Furthermore, it reinforces the evidence that, even though the vast majority of the population now has immune memory toward SARS-CoV-2, early therapeutic strategies aimed at rescuing neutralization of the infecting variant (i.e., next-generation monoclonal antibodies or well-characterized convalescent plasma) may improve survival in both immunocompetent and immunocompromised critically ill COVID-19 patients.

## Supplementary Information

Supplementary Material 1.

## Data Availability

All data supporting the findings of this study are available within the paper and are available from the corresponding author upon request.

## Abbreviations

AAB-I-IFNs: Auto-antibodies against type I interferons
Ct: Cycle threshold
ICU: Intensive care unit
WHO: World Health Organization
SOFA: Sequential Organ Failure Assessment
