# Supplementary material for: Impaired serum neutralization and death in Omicron-infected critically ill patients: insights from the French SEVARVIR prospective, multicenter cohort study
Source: Intensive Care Med Exp. 2025 Nov 26;13:121. doi: 10.1186/s40635-025-00831-y (PMC12657706; doi:10.1186/s40635-025-00831-y)
Supplement: Supplementary file 1 — Supplementary Material 1. [file 40635_2025_831_MOESM1_ESM.docx]

**SUPPLEMENTARY DATA**

**Impaired serum neutralization and death in Omicron-infected critically ill patients**

**Table of content**

Table S1........................................................................................................................Page 2

Table S2........................................................................................................................Page 3

Table S3........................................................................................................................Page 5

**Table S2. List of the 33 selected antigens**

| **Antigens** | **Classification** |
| --- | --- |
| Coronavirus 229E_NP | Respiratory virus |
| Coronavirus 229E_S | Respiratory virus |
| Coronavirus HKU1_NP | Respiratory virus |
| Coronavirus HKU1_S | Respiratory virus |
| Coronavirus NL63_NP | Respiratory virus |
| Coronavirus NL63_S | Respiratory virus |
| Coronavirus OC43_NP | Respiratory virus |
| Coronavirus OC43_S | Respiratory virus |
| EBV_gp125 | Respiratory virus |
| HHV-6B | Respiratory virus |
| Flu-A H1N1_HA1_Michigan_2015 | Respiratory virus |
| Flu-A H3N2_HA_Darwin_2021 | Respiratory virus |
| hMPVA | Respiratory virus |
| hMPVB | Respiratory virus |
| Flu-A | Respiratory virus |
| Rhinovirus_T1A | Respiratory virus |
| RSV-A | Respiratory virus |
| RSV-B | Respiratory virus |
| RSV_gG | Respiratory virus |
| SARS CoV-2 NP | Respiratory virus |
| SARS CoV-2 RBD_Omicron | Respiratory virus |
| SARS CoV-2 RBD_Wuhan | Respiratory virus |
| SARS CoV-2 Spike_Wuhan | Respiratory virus |
| SARS CoV-2 Spike_S2 | Respiratory virus |
| SARS CoV-2 Spike_Omicron | Respiratory virus |
| EBV_gp125 | High-prevalence antigens |
| HHV-6B | High-prevalence antigens |
| Bordetella_p_Toxin | Vaccine antigens |
| Diptheria_Toxin | Vaccine antigens |
| measles_NP | Vaccine antigens |
| Tetanus_Toxin | Vaccine antigens |
| measles_lysate | Vaccine antigens |
| mumps_lysate | Vaccine antigens |
| mumps_NP | Vaccine antigens |
| Rub_VLP | Vaccine antigens |

*NP: nucleoprotein; S: spike; gp : glycoprotein; gG: glycoprotein G; RBD : receptor-binding protein; VLP: Virus-like particle*

**Table S2. Characteristics of critically ill COVID-19 patients exposed to anti-CD20 antibodies (n=9).**

|  | **All**  **(n=9)** |
| --- | --- |
| Age, year | 74 (62-75) |
| Gender, female | 2 (22.2) |
| **Comorbidities** | |
| Diabetes | 0 |
| Chronic heart failure | 2 (22.2) |
| Chronic respiratory failure | 1 (11.1) |
| Chronic renal failure | 2 (22.2) |
| Immunosuppression | 9 (100) |
| *Hematological malignancies* | 8 |
| *Solid organ transplant* | 0 |
| *Solid cancer < 3 years* | 0 |
| *Auto-immune disorders^1^* | 1 |
| *Anti-CD20 monoclonal antibodies* | 9 |
| **Clinical presentation at ICU admission** | |
| First symptoms-ICU admission, days | 3 (2-14) |
| WHO 10-point severity scale | 6 (5-7) |
| SOFA score | 4 (2-6) |
| White blood cell counts, G/L | 2.9 (2.3-14.1) |
| Blood lymphocytes, G/L | 0.2 (0.1-0.5) |
| **SARS-CoV-2 vaccination and infection history** | |
| Complete vaccination* | 5 (55.5) |
| Number of doses of vaccine | 3 (2-4) |
| Proven previous SARS-CoV-2 infection | 1 (11.1) |
| Hybrid immunity** | 1 (11.1) |
| SARS-CoV-2 neutralization titer <15 | 8 (88.9) |
| SARS-CoV-2 RT-PCR, CT | 27 (19-29) |
| Infecting variant |  |
| CH.1.1 | 1 (11.1) |
| BQ.1.1 | 0 (0) |
| XBB.1.5 | 4 (44.4) |
| JN.1 | 4 (44.4) |
| **Outcomes and management during ICU stay** | |
| Organ failures | 5 (55.5) |
| ICU-acquired infection | 5 (55.5) |
| Dexamethasone | 4 (44.4) |
| Tocilizumab | 3 (33.3) |
| Antiviral treatments |  |
| *Convalescent plasma* | 1 (11.1) |
| *Monoclonal antibodies (tixagevimab/cilgavimab)* | 0 |
| *Remdesivir* | 1 (11.1) |
| Day 28 mortality | 8 (88.9) |

*Qualitative variables are shown as n (%) and continuous variables as median (interquartile range 25-75); ^1^Fibrillary glomerulonephritis (n=1), systemic lupus erythematosus (n=1), Anti–glomerular basement membrane antibody disease (n=1), autoimmune hepatitis (n=1), autoinflammatory disease (n=1); ^2^HIV infection (n=1), sickle cell disease (n=1); *3 doses; **at least one vaccine dose received and previous SARS-CoV-2 infection; ECMO: extra corporeal membrane oxygenation.*

**Table S3. Characteristics of critically ill COVID-19 patients with negative (n=13) or positive (n=36) seroneutralization (ancestral variant).**

|  | **Negative seroneutralization (n=13)** | **Positive seroneutralization (n=36)** | | **P value** |
| --- | --- | --- | --- | --- |
| Age, year | 73 (50-76) | 72 (61-76) | | 0.77 |
| Gender, female | 5 (38.5) | 12 (33.3) | | 0.74 |
| SOFA score | 4 (3-6) | 5 (2-7) | | 0.13 |
| **Comorbidities** | | | | |
| Diabetes | 8 (28.6) | 4 (19.0) | | 0.82 |
| Chronic heart failure | 2 (15.4) | 6 (16.7) | | 0.91 |
| Chronic respiratory failure | 2 (15.4) | 9 (25.0) | | 0.48 |
| Chronic renal failure | 2 (15.4) | 8 (22.2) | | 0.60 |
| Immunosuppression* | 8 (61.5) | 18 (50.0) | | 0.47 |
| *Haematological malignancies* | 5 | 5 | |  |
| *Solid organ transplant* | 2 | 2 | |  |
| *Solid cancer < 3 years* | 0 | 5 | |  |
| *Auto-immune disorders^1^* | 1 | 4 | |  |
| *Anti-CD20 in last 12 months* | 5 (38.5) | 4 (11.1) | |  |
| *Others^2^* | 0 | 2 | |  |
| **Vaccination and infection history** | | | | |
| First symptoms-ICU admission, days | 4.0 (1.0-11.0) | | 5 (3.0-8.7) | 0.63 |
| Complete vaccination** | 7 (53.8) | | 23 (76.7) | 0.50 |
| Number of doses of vaccine | 3 (0-4) | | 3 (3-4) | 0.22 |
| Previous SARS-CoV-2 infection | 2 (15.4) | | 5 (13.5) | 0.87 |
| Hybrid immunity*** | 2 (15.4) | | 5 (13.5) | 0.87 |
| SARS-CoV-2 neutralization titer | <15 | | 1521 (529-14454) | **<0.0001** |
| SARS-CoV-2 RT-PCR, CT | 19 (16-24) | | 21 (17-25) | 0.67 |
| **Outcomes and management** | | | | |
| Organ failures | 5 (38.5) | | 17 (47.2) | 0.59 |
| Invasive mechanical ventilation | 4 (30.8) | | 14 (38.9) | 0.60 |
| ICU-acquired infection | 1 (7.7) | | 4 (11.1) | >0.99 |
| Dexamethasone | 8 (61.5) | | 21 (58.3) | 0.84 |
| Tocilizumab | 3 (23.1) | | 6 (16.7) | 0.60 |
| Day 28 mortality | 4 (30.8) | | 6 (16.7) | 0.28 |

*Qualitative variables are shown as n (%) and continuous variables as median (interquartile range 25-75); ^1^Negative seroneutralization: Fibrillary glomerulonephritis (n=1), systemic lupus erythematosus (n=1), Anti–glomerular basement membrane antibody disease (n=1); Positive seroneutralization: autoimmune hepatitis (n=1), autoinflammatory disease (n=1); ^2^HIV infection (n=1), sickle cell disease (n=1); *Patients can have one or more cause of immunosuppression; **At least 3 doses received (vaccination status was missing for five patients); ***at least one vaccine dose received and previous SARS-CoV-2 infection; P values come from Chi square or Fisher’s exact tests for categorical variables, and Mann-Whitney tests for continuous variables, as appropriate;* ***Bolded*** *values are significant at the < 0.05 level. Neutralizing titer is measured against the infecting variant.*
